# VEGF-D-induced draining lymphatic enlargement and tumor lymphangiogenesis promote lymph node metastasis in a xenograft model of ovarian carcinoma

**DOI:** 10.1186/1477-7827-12-14

**Published:** 2014-02-06

**Authors:** Li-Cheng Du, Xian-Cheng Chen, Dong Wang, Yan-Jun Wen, Chun-Ting Wang, Xue-Mei Wang, Bing Kan, Yu-Quan Wei, Xia Zhao

**Affiliations:** 1 Department of Surgery, Shandong Provincial Hospital affiliated to Shandong University, Jingwu Road, Jinan, China; 2 Department of Gynecology and Obstetrics, Second West China Hospital, West China Medical School, Sichuan University, South Renmin Road, Chengdu, China; 3 National Key Laboratory of Biotherapy and Cancer Center, West China Hospital, West China Medical School, Sichuan University, South Renmin Road, Chengdu, China

**Keywords:** Ovarian carcinoma, VEGF-D, Metastasis, Lymphangiogenesis, Xenograft

## Abstract

**Background:**

Vascular endothelial growth factor (VEGF)-D has been shown to promote lymph node metastasis in several cancers. Although generally overexpressed in ovarian carcinoma, its role in nodal dissemination of this cancer is unclear. To clarify the role of VEGF-D and the underlying molecular mechanisms, we investigated the function of VEGF-D using a mouse xenograft model of ovarian cancer.

**Methods:**

Human ovarian serous adenocarcinoma SKOV3 cells were transfected with VEGF-D recombinant plasmid DNA, or with control vectors. The cells were injected subcutaneously into the footpads of nude mice. Tumor growth was evaluated weekly. Draining lymphatics were observed grossly with Evan’s blue lymphangiography. Tumoral lymphatics were delineated with both Evan’s blue and LYVE-1 immunostaining. Tumor metastases to lymph nodes were evaluated by H&E and CA125/CD40 staining. Expression of VEGF-D in primary tumors and levels of CA125 in involved lymph nodes were examined by immunohistochemistry. Tumor cell apoptosis was analyzed by Hoechst dyeing.

**Results:**

Mice bearing VEGF-D overexpressing xenografts showed a significantly higher rate of lymph node metastasis and markedly greater tumor volume compared with the controls. The functional lymphatic vessels were denser and enlarged in marginal and central tumor portions. Additionally, higher CA125 expression was observed in the involved lymph nodes. Mice bearing VEGF-D overexpressing xenografts also exhibited a markedly lower apoptotic index compared with the controls.

**Conclusions:**

Our data demonstrate the important role of VEGF-D in promoting lymph node metastasis by increasing tumor lymphangiogenesis, stimulating draining lymphatic vessel formation, and enhancing tumor invasiveness. Our findings show that VEGF-D can be a promising therapeutic target for ovarian cancer.

## Background

Lymph node metastasis is a major biological characteristic of ovarian malignancies and is commonly seen in advanced stage ovarian cancer with an incidence of 67.2% in para-aortic and pelvic lymph node metastasis. For early stage ovarian cancer, lymph node metastasis is seen in 10% to 30% of the cases [1,2]. Lymph node status is an important predictor of poor prognosis in patients with ovarian carcinoma [3-5]. Patients with positive lymph node have a significantly shorter overall survival than node-negative patients [1]. Moreover, recent investigations have shown that the absence of lymph node metastases is associated with lack of distant organ metastases, highlighting a critical role of lymph node metastasis in tumor dissemination [6]. Currently, there are no effective therapeutic strategies against lymph node metastasis. Pelvic and para-aortic lymphadenectomy removes involved nodes, but is not recommended for early-stage ovarian cancer patients due to complications and associated morbidities [7,8].

The finding of vascular endothelial growth factors (VEGF) mediating lymphangiogenesis and thereby promoting lymphatic metastasis in tumors provides a new therapeutic target. Of all the prolymphangiogenic factors, VEGF-D is a major effector. VEGF-D, also known as c-fos-induced growth factor, is a secreted growth factor consisting of VEGF-homology domain, receptor binding domains, and propeptides in both termini. After secretion into the extracellular space, the *C*– and *N*-terminal propeptides are cleaved from full length VEGF-D to form mature VEGF-D. This proteolytic processing increases the affinity of VEGF-D for VEGFR-3, a tyrosine kinase receptor that is mainly located on adult lymphatic endothelium and is implicated in lymphangiogenesis [9,10]. VEGF-D also interacts with a non-tyrosine kinase receptor Nrp-2, another lymphangiogenesis-associated factor [11]. A prominent role of VEGF-D in lymph node metastasis is supported by findings from several studies of cancer [12-15], but not by others [16-18].

In patients with epithelial ovarian carcinoma, VEGF-D expression has been shown to be a predictor of poor outcome [3,4]. We hypothesized that VEGF-D may be a potent promoter of lymphatic metastasis of ovarian cancer cells. In the current study, we examined the potential effects of VEGF-D overexpression on ovarian carcinoma growth and lymphangiogenesis by using a mouse model bearing human ovarian carcinoma xenografts that overexpress VEGF-D.

## Methods

### Mice

Female athymic nude mice (7–8 weeks of age, 18–20 grams) were obtained from the Animal Center of Sichuan University, Sichuan, China and housed in environmentally controlled conditions (22°C, a 12 h light/dark cycle with the light cycle from 6:00 to 18:00) with *ad libitum* access to standard rodent chow and water. The study protocol was approved by the local institutional review board at the authors’ affiliated institution and all animal experiments were performed in accordance with the National Animal Care and Use Guidelines of the US National Institute of Health (NIH).

### Generation of cell line stably expressing VEGF-D

Human epithelial serous cystadenocarcinoma SKOV3 cells (American Type Culture Collection, Manassas, VA) were grown in RPMI-1640 with 10% fetal bovine serum. The cells were transfected with a vector containing a mouse VEGF-D cDNA (GenBank NM_010216 GI: 6753873) or with the control vector pcDNA3.1(+) (Invitrogen, Carlsbad, CA). Stable cells expressing VEGF-D, designated SKOV3/VEGF-D, or control cells, designated SKOV3/pcDNA were established after selection with 50 μg/mL geneticin (Sigma, St. Louis, MO).

### RT-PCR

Total RNA was prepared from 1 × 10^7^ cells. First strand cDNA was synthesized and then amplified with PCR consisting of the following cycles: 94°C for 30 s; 55°C for 30 s; and 72°C for 1.5 min for a total of 30 cycles. The *VEGF-D* gene was amplified using the following primers: 5′-GCAAGCTTATGTATGGAGAATGGGGAATG-3′, sense, and 5′-CGTCTAGATCAAGGGTTCTCCTGGCTG-3′, antisense. The PCR products were visualized by gel electrophoresis. The expression of VEGF-D was normalized against β-actin.

### Animal experiments

Totally, 1.5 × 10^6^ cells (suspended in 50 μL serum-free medium) were injected into the left hind-footpads of mice. The experiments were carried out for three times independently with six mice in each group. Tumor growth and sentinel lymph nodes were evaluated weekly. Tumor volume was determined using the following formula: tumor volume (mm^3^) = 0.52 × length (mm) × width (mm) × height (mm). Mice were sacrificed after 12 to 15 weeks. Lymph nodes, labeled by their locations, as well as primary tumors, were collected, fixed in formalin, and embedded in paraffin.

### Lymphatic vessel discrimination

Lymphatic vessels were grossly observed using Evan’s blue. Briefly, before the mice were sacrificed, Evan’s blue was injected subcutaneously into the tumors of the footpads until the inguinal lymph nodes were marked. Then, an additional 5 μL of the dye was injected. The draining lymphatic vessels of inguinal trunk along the milk line were scored as: negative (-) if not visualized or vague, and positive (+) if clearly seen and enlarged.

Microscopic lymphatic vessel estimation was performed by immunostaining with antibody against LYVE-1 as detailed below. The LYVE-1-positive lymphatic vessels were counted microscopically under 400× magnification in three separate areas of the highest vascular density (vascular 'hot spots’) per Section. A comparative analysis of the number of lymphatic vessels in intra- and peri-tumoral areas was performed.

### Immunostaining

Mice were sacrificed after 12 to 15 weeks. Lymph nodes, labeled by their locations, as well as primary tumors, were collected, fixed in formalin, and embedded in paraffin. Immunostaining of VEGF-D, LYVE-1, CA125, and CD40 was performed using a streptavidin-biotin-peroxidase complex method (Boster Bio, Wuhan, China). The following primary antibodies were used: rat anti-mouse VEGF-D monoclonal antibody (R&D Systems), goat anti-mouse polyclonal LYVE-1 antibody (Santa Cruz Biotechnology, Santa Cruz, CA), mouse anti-human CA125 monoclonal antibody (Zhongshan-Golden Bridge, Beijing, China), and mouse anti-human CD40 monoclonal antibody (Santa Cruz Biotechnology). Biotin-conjugated secondary antibodies were from Boster Bio. Sections were counterstained with hematoxylin. Background binding was determined by omission of the primary antibody.

VEGF-D and CA125 immunostaining was evaluated under a light microscope independently by two experienced pathologists. VEGF-D and CA125 reactivity was defined as granular and diffuse cytoplasmic, membranous or nuclear staining and evaluated according to intensity and proportion. The intensity of staining was scored as follows: negative (±), 0-25% positive cells; weak (+), >25–50% positive cells; moderate (++), >50–75% positive cells; strong (+++), >75% positive cells. The photographs were taken using a Leica digital microscope color camera (DFC295, Germany) at 20×-magnification of objective lens by Leica light microscope (DM2500, Germany) with a total magnification of 400×.

### Evaluation of lymphatic metastasis

Lymph nodes were harvested, fixed in formalin, and embedded in paraffin. Each lymph node was labeled with its location: popliteal, parailiac, renal hilum, inguinal, or axillary node. Metastases were examined by hematoxylin and eosin (H&E) staining. Atypical morphological metastasis was further verified by CA125 or CD40 immunostaining [19-21]. Lymph node metastases were assessed by two pathologists in a blinded manner, and expressed as a ratio of metastatic lymph nodes out of the total number of lymph nodes.

### Apoptosis analysis

Tissue sections were deparaffinized and rehydrated through xylene, gradient ethanol, and distilled water. After rinsing three times in PBS, the tissue sections were incubated with 1 μmol/L Hoechst-33258 (Sigma) for ten minutes. After washing three times in PBS, slides were transferred to coverslip with antifade solution (Sigma), and then analyzed by fluorescence microscopy. Cells with condensed chromatin or fragmented nuclei emitting intense blue fluorescence were classified as apoptotic.

### Statistical analysis

Statistical analyses were carried out with the SPSS software version 15.0 (SPSS Inc., Chicago, IL). The relationship of VEGF-D with lymph node metastasis was evaluated using Fisher’s exact or Pearson Chi-Square tests. Student-Newman-Keuls test was used to examine homogeneity. Tumor volume, lymph vessel density and apoptosis index were analyzed using univariate analysis of variance. Comparison of ranked data (e.g., VEGF-D or CA125 expression) was performed using Kruskal-Wallis Test. Continuous variables are given as mean ± SD. *P* < 0.05 was considered statistically significant.

## Results

### VEGF-D overexpression promotes growth of ovarian cancer xenografts

**Figure 1:**
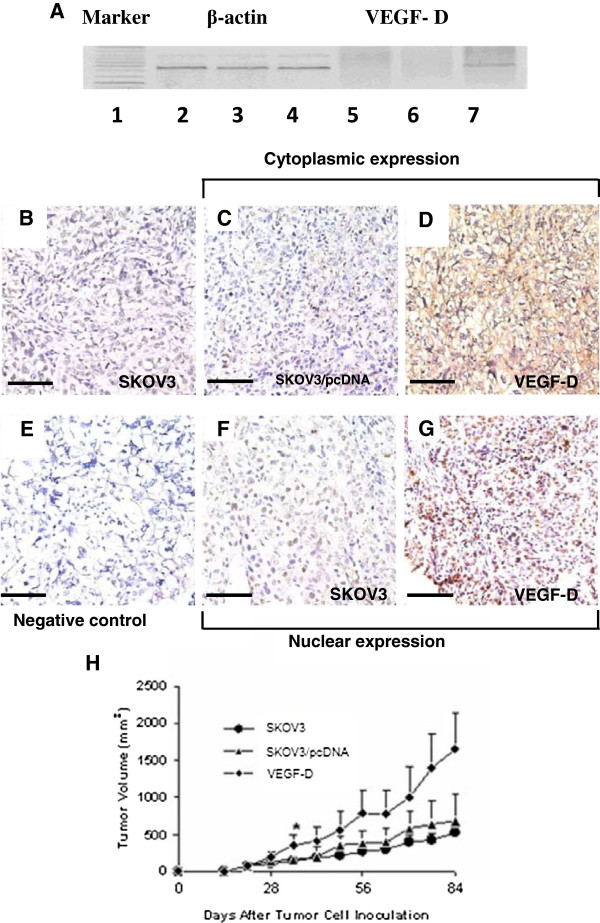
**VEGF-D overexpression promotes mouse xenograft growth. (A)** RT-PCR assays reveal the presence of VEGF-D mRNA transcripts only in SKOV3 cells stably expressing VEGF-D (lane 7), not in SKOV3 cells (lane 5) or SKOV3 cells transfected with control vectors (lane 6). β-actin was used as a loading control (lane 2, SKOV3 cells, lane 3, SKOV3 cells transfected with control vector, and lane 4, SKOV3 cells stably expressing VEGF-D). **(B-G)** Immunostaining shows that VEGF-D is strongly expressed in the cytoplasm and nuclei of tumor cells stably expressing VEGF-D from mouse xenografts **(D and G)** while it is only weakly expressed in SKOV3 cells or SKOV3 cells transfected with control vectors **(B, C and F)**. Magnification: ×400, scale bar = 200 μm. **(H)** Mice were inoculated subcutaneously with SKOV3, SKOV3 /pcDNA or SKOV3/VEGF-D cells. Tumor growth was monitored by measuring tumor volume post inoculation. Data are expressed as mean ± SD of three independent experiments. *P < 0.05 versus tumor xenografts bearing SKOV3 or SKOV3 transfected with control vectors.

Efficiency and selectivity of the vectors was verified by RT-PCR (Figure 1A). Immunohistochemistry showed that mouse xenografts bearing SKOV3/VEGF-D cells exhibited apparent strong staining of VEGF-D in both the cytoplasm and nuclei of the tumor cells while only weak or negative staining of VEGF-D was observed in mouse xenografts bearing SKOV3 cells or SKOV3/pcDNA cells (Figure 1B to 1G).

Xenografts bearing SKOV3 cells overexpressing VEGF-D grew at an apparent faster pace from week 3 post inoculation compared to xenografts bearing SKOV3 cells or SKOV3 cells transfected with control vectors. By week 5, the tumor volume in VEGF-D overexpressing xenograft was significantly greater than that of xenografts bearing SKOV3 cells or SKOV3 cells transfected with control vectors (P < 0.05) (Figure 1H).

### VEGF-D overexpression promotes lymphangiogenesis in mouse xenografts

**Figure 2:**
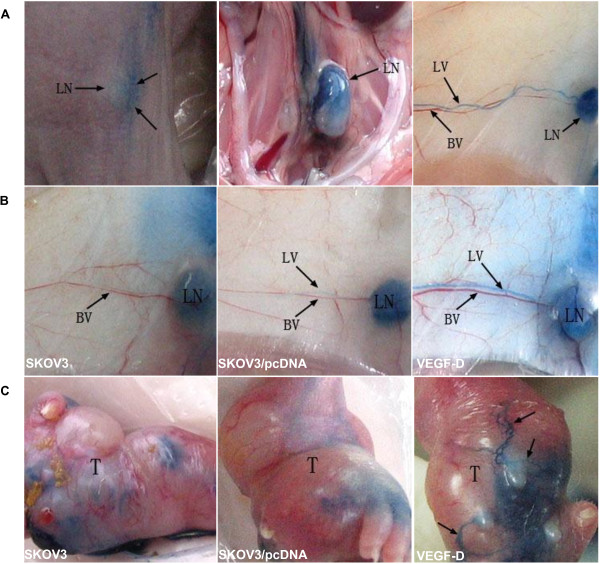
**VEGF-D promotes lymphangiogenesis. (A)** Footpads bearing tumor xenografts are injected with Evan’s blue dye, which appears in the lymph nodes (LNs) and lymphatic vessels (LVs). No Evans blue is observed in the blood vessels (BVs) and other adjacent tissues. **(B)** In the drainage route from the inguinal to the axillary LN, mice bearing xenografts overexpressing VEGF-D show dilated LVs (right panel), while LVs in mice bearing SKOV3 (left panel) or SKOV3/pcDNA (mid panel) xenografts show no apparent dilation. **(C)** LVs are not apparent on the tumor surface in mice bearing SKOV3 (left panel) or SKOV3/pcDNA (mid panel) xenografts. LVs are apparent and enlarged and form new networks (arrows) on the surface of tumors overexpressing VEGF-D.

In comparison to mouse xenografts bearing SKOV3 cells or SKOV3 cells transfected with control vectors, xenografts bearing SKOV3 cells overexpressing VEGF-D showed grossly dilated lymphatic vessels (Figure 2). Lymphatic vessels were not prominent on the tumor surface in mice bearing SKOV3 cells or SKOV3 cells transfected with control vectors. In contrast, the lymphatic vessels were apparent and enlarged, with newly formed lymphatic networks on the surface of tumors overexpressing VEGF-D (Figure 2C and Table 1).

**Table 1:** Gross staining by Evan’s blue dye of the inguinal trunk in mice bearing ovarian cancer xenografts

	**Negative**	**Positive**
*SKOV3*	6/6	0/6
*SKOV3/pcDNA*	5/6	1/6
*SKOV3/VEGF-D*	1/6	5/6*

*P < 0.05 vs. mice bearing SKOV3 or SKOV3/pcDNA xenografts.

**Figure 3:**
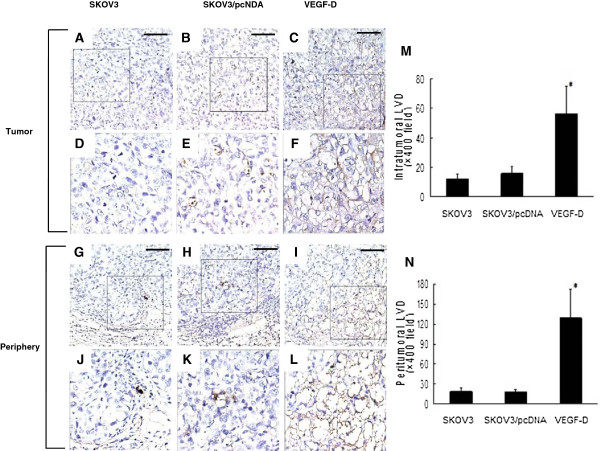
**VEGF-D overexpression promotes lymphangiogenesis. (A-L)** Expression of LYVE-1 within and at the periphery of tumor tissues is examined by immunohistochemistry. The region with the highest lymphatic vessel density is dotted in the black box **(A-C and G-I)** and shown at higher magnification in **D**-**F** and **J**-**L**. Mean lymphatic vessel density ± SD of three independent experiments are shown in **M** and **N**. *P < 0.01 vs. mice bearing SKOV3 or SKOV3/pcDNA xenografts. Original magnification: ×400. Scale bar = 200 μm in **A-C** and **G-I**.

Immunostaining with an antibody against LYVE-1 (a highly selective marker for lymphatic vessels) showed numerous lymphatic vessels with open lumen in the center of SKOV3 xenograft overexpressing VEGF-D, and only sparse lymphatic vessels in SKOV3 or SKOV3/pcDNA xenograft (Figure 3A to F). More significant difference was found at the tumor margin (Figure 3G to L). The lymphatic vessel density in VEGF-D overexpressing xenografts was much higher (55.83 ± 18.73 vs. 12.00 ± 3.46 for SKOV3 or15.83 ± 4.40 for SKOV3/pcDNA xenografts; *P* < 0.001 for both) (Figure 3M). The lymphatic vessel density at the margin of VEGF-D overexpressing tumors was also significantly higher (128.67 ± 42.78 vs. 18.67 ± 5.35 for SKOV3 or17.17 ± 5.00 for SKOV3/pcDNA xenografts; *P* < 0.001 for both) (Figure 3N).

### VEGF-D overexpression potentiates lymphatic metastasis of ovarian cancer cells

**Figure 4:**
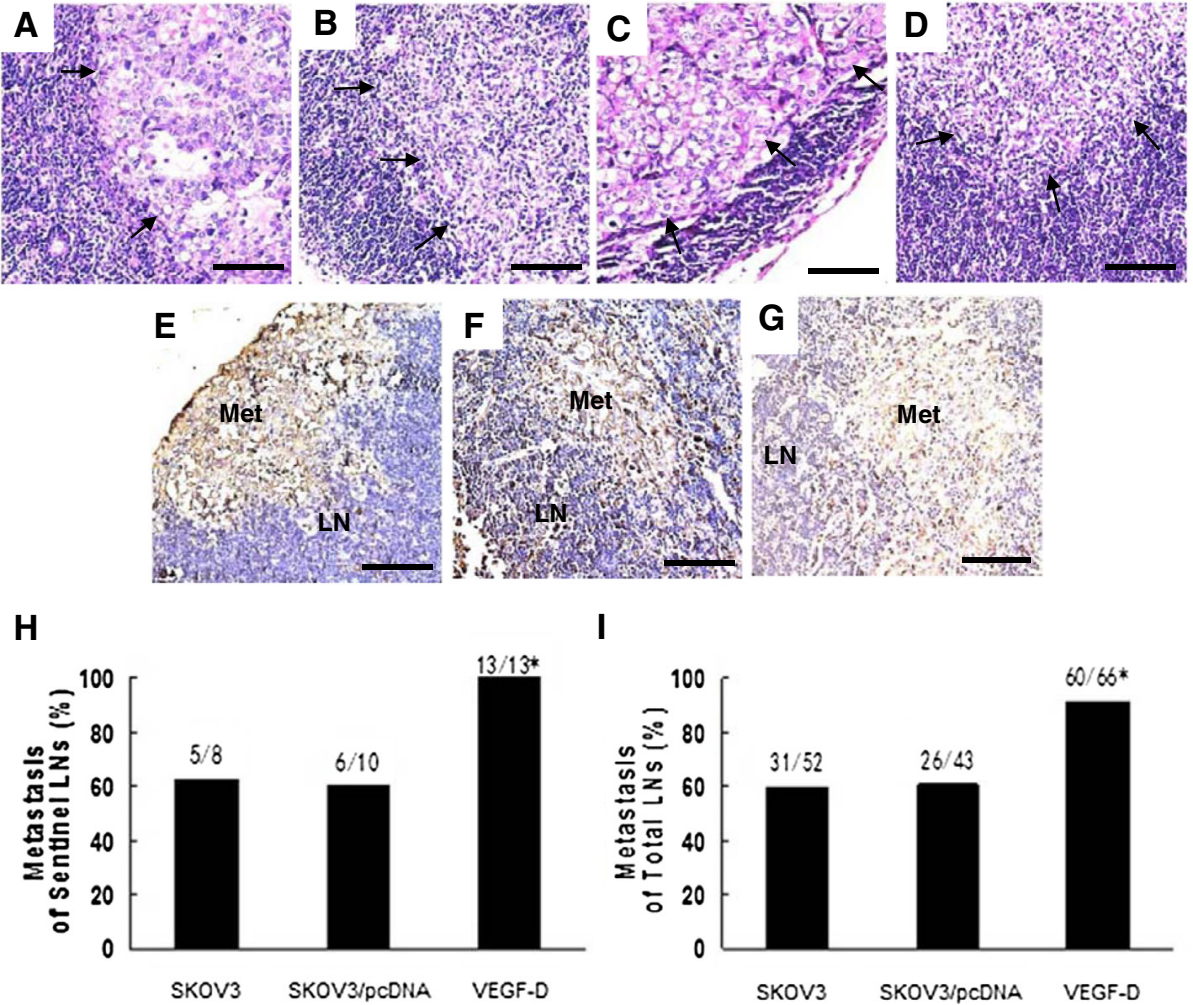
**VEGF-D overexpression potentiates lymphatic metastasis of ovarian cancer cell. (A-D)** Histologic examination of lymph nodes in the renal hilum reveals the presence of metastasis (indicated by arrows). **(A)** and **(B)** are different fields of view of the same section, and so are **(C)** and **(D)**. **(E-G)** Lymph nodes are immunostained for CA125 **(E and F)** and CD40 **(G)**. **(H-I)** Percentage of lymph nodes with metastasis in the sentinel or total lymph nodes is shown. *P < 0.05 vs. xenografts bearing SKOV3 or SKOV3/pcDNA cells in the sentinel nodes. *P < 0.01 vs. xenografts bearing SKOV3 or SKOV3/pcDNA cells in total lymph nodes. Original magnification: ×400; scale bar = 200 μm. LN, lymph nodes; Met, metastasis.

Metastasis in the lymph nodes of mice bearing ovarian cancer cells was apparent in H&E staining of lymph node tissue sections (Figure 4A to D), as well as immunostaining for CA125 or CD40 (Figure 4E to G). Furthermore, 100% (13/13) of the examined sentinel lymph nodes in mice bearing VEGF-D overexpressing xenografts contain tumor cells, whereas the rate of metastasis to sentinel lymph node was only 62.5% (5/8) and 60% (6/10) in mice bearing SKOV3 or SKOV3/pcDNA xenografts, respectively (*P* < 0.05 for both; Figure 4H). In an analysis that included all lymph nodes, the metastasis rate was 90.9% (60/66) in mice bearing VEGF-D overexpressing xenografts in contrast to 59.6% (31/52) and 60.5% (26/43) in mice bearing SKOV3 or SKOV3/pcDNA xenografts, respectively (*P* < 0.01 for both; Figure 4I).

### VEGF-D overexpression upregulates CA125 in the metastatic lymph nodes

**Figure 5:**
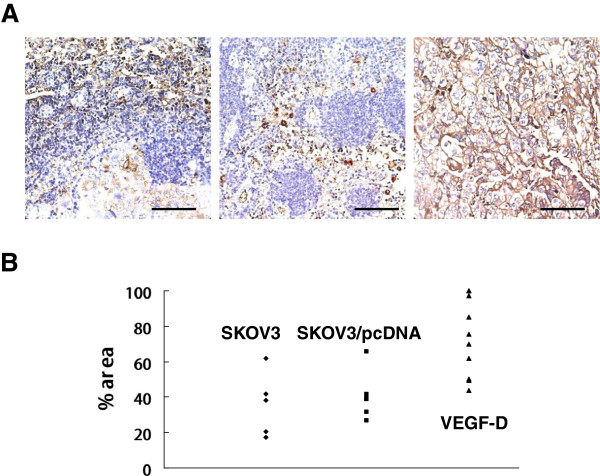
**VEGF-D overexpression upregulates CA125 in the metastatic lymph nodes. (A)** Representative sections for CA125 in lymph nodes of mice bearing SKOV3 xenograft- (left panel), SKOV3/pcDNA xenograft (mid panel) or SKOV3/VEGF-D xenografts (right panel). **(B)** Percentage of CA125-positive area. *P* < 0.01 vs. mouse bearing SKOV3 or SKOV3/pcDNA xenografts. Each data point represents a single lymph node. Original magnification: ×400; scale bar = 200 μm.

CA125 expression was markedly enhanced in the sentinel lymph nodes of mice bearing VEGF-D overexpressing xenografts in comparison to that in mice bearing SKOV3 or SKOV3/pcDNA xenografts (Figure 5A). Many lymph nodes in mice bearing VEGF-D overexpressing xenografts were almost entirely occupied by CA125-positive tumor cells (Figure 5A, right panel). The percentage of CA125-positive area in the sentinel lymph nodes of mice bearing VEGF-D overexpressing xenografts was approximately 2-fold higher than that of mice bearing SKOV3 or SKOV3/pcDNA xenografts (P < 0.01 for both; Figure 5B). The percentage of the sentinel lymph nodes strong positive for CA125 (++ or higher) was 69.2% (9/13) in mice bearing xenografts overexpressing VEGF-D in contrast to 0% and 16.7% in mice bearing SKOV3 xenografts or SKOV3/pcDNA xenografts (*P* < 0.01 for both; Table 2).

**Table 2:** CA125 staining intensity in the metastatic lymph nodes

	**±**	**+**	**++**
*SKOV3*	3/5	2/5	0/5
*SKOV3/pcDNA*	2/6	3/6	1/6
*SKOV3/VEGF-D*	1/13	3/13	9/13*

*P < 0.01 vs. mice bearing SKOV3 or SKOV3/pcDNA xenografts.

### VEGF-D inhibits apoptosis of ovarian cancer cells

**Figure 6:**
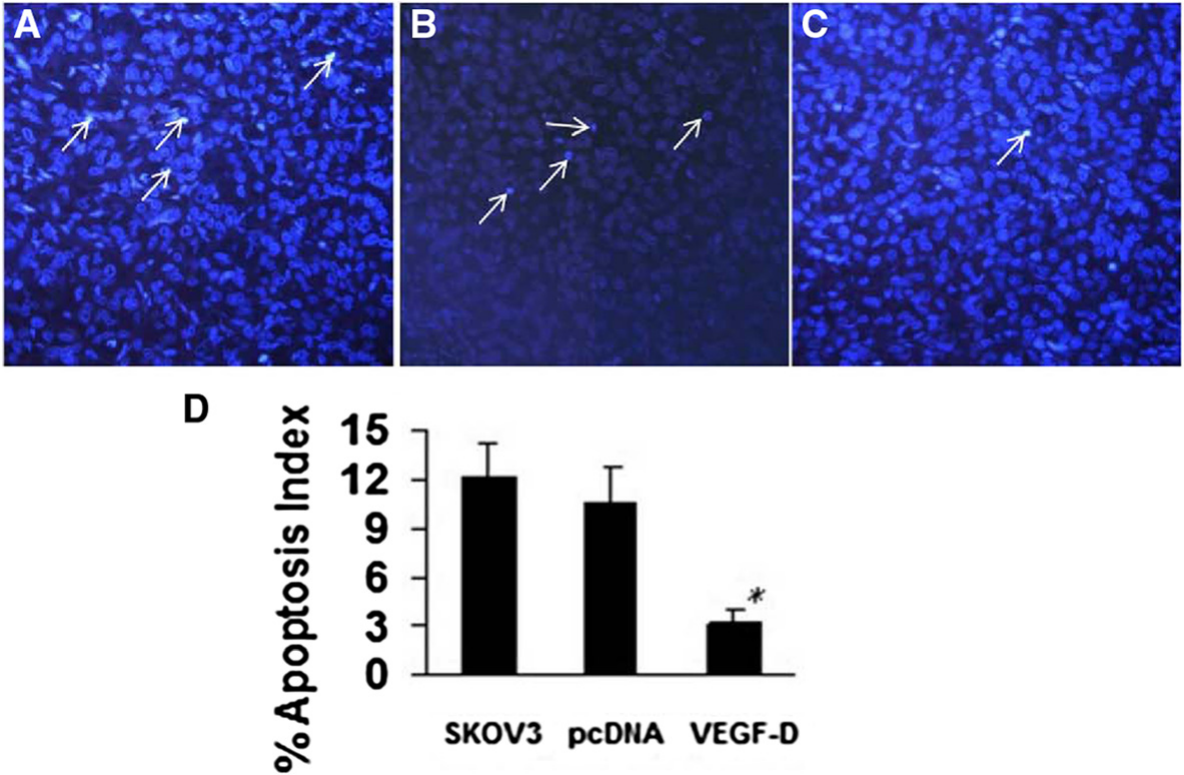
**VEGF-D overexpression suppresses apoptosis of ovarian cancer cells.** Tumor tissues were stained with Hoechst 33258. Apoptotic cells are indicated by arrows **(A-C)**. **(A)** Tumors bearing SKOV3 cells; **(B)** tumors bearing SKOV3/pcDNA cells; **(C)** tumors bearing SKOV3/VEGF-D cells. **(D)** Apoptosis index of tumor cells. *P* < 0.05 vs. xenografts bearing SKOV3 or SKOV3/pcDNA.

There was no difference in the apoptotic rate between xenografts bearing SKOV3 (12.2 ± 1.94%) and xenografts bearing SKOV3/pcDNA (10.6 ± 2.15%) (Figure 6A and B). VEGF-D overexpressing xenografts showed a significantly lower apoptotic index (3.2 ± 0.748%; *P* < 0.05) (Figure 6C and D).

## Discussion

Tumor metastasis to regional lymph nodes often represents the first step of tumor dissemination, which precedes metastasis via the vascular system and serves as a major prognostic indicator of tumor progression [22-24]. VEGF–C and VEGF-D act predominantly via their receptor VEGFR-3, which is largely restricted to the lymphatic endothelium in normal adult tissue [22]. VEGF-C level correlates with active lymphangiogenesis, increases lymphatic tumor spread to regional lymph nodes [25,26], and decreases with decreasing activity of the VEGF-C/VEGFR-3 signaling pathway [27,28].

VEGF-D could induce tumor-associated lymphangiogenesis, and by doing so, promotes lymphatic spread [4,12,13,29-31]. Such a notion was not supported by several studies on breast cancer showing that VEGF-D has no demonstrable effect on lymphangiogenesis or lymphatic metastasis [18,32,33]. Niki *et al.* showed an inverse correlation between VEGF-D and lymphogenous metastasis in lung adenocarcinoma [16]. The role of VEGF-D in lymphangiogenesis and lymphatic spread of ovarian carcinoma remains controversial [3,4,34].

Our study shows that VEGF-D could induce tumor lymphangiogenesis and increase the spread of ovarian carcinoma cells to the sentinel and upper-grade lymph nodes. This is likely due to the activation of VEGFR-3 by VEGF-D on the lymphatic endothelium in tumors. Lymphatic vessel density is found to be higher in cancer patients with nodal involvement than those without [35]. In our study, lymphatic vessels were clearly seen both within and at the periphery of tumors, and the lymphatic vessel density in VEGF-D-transfected tumors was much higher than that in the controls. The lower lymphatic vessel density in the central area of tumors compared to the peripheral area may be due to higher intra-tumoral interstitial pressure.

Many investigations have focused on the interaction between VEGF-D and tumor lymphangiogenesis, or lymphangiogenesis within lymph nodes [36]. However, few studies have addressed the role of VEGF-D on lymphatics. In this study, we used Evan’s blue to examine the lymphatic networks in mouse xenografts of ovarian cancer. Intriguingly, we observed that the draining lymphatic network was greatly enhanced in mice bearing xenografts overexpressing VEGF-D. This finding suggests that VEGF-D overexpression not only promotes intratumoral lymphangiogenesis but also enhances extratumoral lymphangiogenesis.

Lymph drainage from the inguinal to the axillary lymph node, named the inguinal trunk in rats [37], was occasionally observed in healthy mice [38]. In the current study, lymphatic vessel enlargement was noticed in 5 out of 6 VEGF-D overexpressing mice. The activation and enlargement of this originally inefficient vessel system is attributable to the overexpression of VEGF-D, and can be explained as a result of VEGFR-3 activation by increased VEGF-D on the lymphatic endothelium [39,40]. This interesting finding, therefore, indicates a novel mechanism for VEGF-D in tumor dissemination in ovarian carcinoma by activating and promoting somatic lymphatic systems, in addition to increasing lymphangiogenesis within the tumors. This novel link between dilation of the collecting lymphatic vessels and lymphatic metastasis was also found in breast cancer, as recently reported by Karnezis *et al*. [41]. Further investigations are required to elucidate this novel mechanism.

In addition to affecting lymphatic endothelial cells, the role of VEGF-D as an autocrine factor for tumor cells have been found in some cancers. VEGF-D has been linked to the CXCL7/CXCR2 axis and tumor cell invasion in a study on breast cancer [42]. In and *in vitro* study of papillary thyroid carcinoma, VEGF-D was found to promote filopodia formation as well as cancer cell migration and invasion [43]. Based on the results from the current study, this autocrine mode apparently is also the case for tumor metastasis to lymph nodes. Upregulated VEGF-D was associated with increased intensity and distribution of ovarian cancer biomarker CA125 in the involved lymph nodes. CA125 has been used as a tumor marker of ovarian carcinoma over the last three decades. It is evaluated for monitoring response to treatment, detecting recurrent disease, distinguishing malignant from benign pelvic masses, and for early detection of ovarian carcinoma. Recently, CA125 has been found to be associated with an invasive phenotype and the metastatic potential of ovarian cancer cells, showing its functional role as an aggressive biomarker for ovarian carcinoma [19,44]. In the current study, CA125 staining was enhanced in the lymph nodes of mice bearing xenografts overexpressing VEGF-D, indicating the role of VEGF-D in promoting tumor cell invasion.

The lymphatic endothelium by far is the only site where VEGFR-3 is reported to be located, suggesting that VEGF-D mainly exerts its action via lymphatics. Our finding that VEGF-D promoted tumor growth *in vivo* could not be readily explained by interaction of VEGF-D with VEGFR-3. We also found that VEGF-D suppressed the apoptosis of ovarian carcinoma cells, suggesting that increased growth of tumor size may be the consequence of subdued apoptosis of the tumor cells. In addition, increased growth of ovarian carcinoma cells further aggravated lymph node metastasis as tumor size has been regarded as a stimulating factor for nodal invasion [45-48]. Consistently, Akahane *et al.* also found that VEGF-D inhibited apoptosis of breast cancer cells [49]. We found more apparent angiogenesis in the tumor tissues overexpressing VEGF-D (data not shown), suggesting its contribution to accelerated tumor growth [50,51].

Lymph node metastasis involves numerous factors. The current study suggested that this process could be initiated and regulated by VEGF-D expressed by tumors. Overexpression of this factor alone is sufficient to promote tumor dissemination to the lymph vessel system. Considering that VEGF-D is overexpressed in human ovarian carcinoma, the metastasis progression in our investigation may mimic the metastatic process in human ovarian cancer. We also investigated the molecular mechanism of VEGF-D in promoting lymphogenous metastasis. In addition to stimulating tumor lymphangiogenesis, VEGF-D unexpectedly stimulated somatic draining lymphatic vessels. This finding implicates VEGF-D in dilating draining lymphatics as a gateway in tumor cell dissemination.

## Conclusions

In conclusion, the current study demonstrated that VEGF-D promotes lymphatic metastasis in ovarian cancer. The findings represent a previously under-appreciated role of VEGF-D in ovarian cancer dissemination, and encourage targeted treatment of ovarian cancer via VEGF-D-mediated pathway.

## Abbreviations

BV: Blood vessel; H&E: Hematoxylin and eosin; IHC: Immunohistochemistry; LN: Lymph node; LV: Lymphatic vessel; LVD: Lymphatic vessel density; LYVE-1: Lymphatic vessel endothelial hyaluronan receptor; RT-PCR: Real-time polymerase chain reaction; SKOV3: Non-transfected SKOV3 cells; SKOV3/PcDNA: SKOV3 cells transfected with control vector pcDNA; SKOV3/VEGF-D: SKOV3 cells transfected with recombinant VEGF-D plasmid DNA; VEGF-D: Vascular endothelial growth factor-D; VEGFR-3: Vascular endothelial growth factor receptor-3.

## Competing interests

The authors declare that they have no competing interests.

## Authors’ contributions

LCD: study design, literature research, experimental studies, data acquisition, data analysis, statistical analysis, manuscript preparation. XCC: literature research, experimental studies, data acquisition, data analysis, statistical analysis, manuscript preparation. DW: literature research, data analysis; statistical analysis; manuscript preparation. YJW: study design, literature research. CTW: *in vitro* experiments, manuscript preparation. XMW: data analysis, statistical analysis. BK: cell culture and inoculation. YQW: study concepts, study design. XZ: guarantor of integrity of the entire study, study concepts, study design, manuscript review. All authors read and approved the final manuscript.
